# Altitudinal Variations of Ground Tissue and Xylem Tissue in Terminal Shoot of Woody Species: Implications for Treeline Formation

**DOI:** 10.1371/journal.pone.0062163

**Published:** 2013-04-26

**Authors:** Hong Chen, Haiyang Wang, Yanfang Liu, Li Dong

**Affiliations:** 1 Institute of Landscape Ecology of Mountainous Horticulture, Southwest University, Chongqing, China; 2 Department of Botany, College of Horticulture and Landscape, Southwest University, Chongqing, China; University of Florida, United States of America

## Abstract

1. The terminal shoot (or current-year shoot), as one of the most active parts on a woody plant, is a basic unit determining plant height and is potentially influenced by a variety of environmental factors. It has been predicted that tissues amount and their allocation in plant stems may play a critical role in determining plant size in alpine regions. The primary structure in terminal shoots is a key to our understanding treeline formation. The existing theories on treeline formation, however, are still largely lacking of evidence at the species level, much less from anatomy for the terminal shoot.

2. The primary structures within terminal shoot were measured quantitatively for 100 species from four elevation zones along the eastern slope of Gongga Mountain, southwestern China; one group was sampled from above the treeline. An allometric approach was employed to examine scaling relationships interspecifically, and a principal components analysis (PCA) was performed to test the relation among primary xylem, ground tissue, species growth form and altitude.

3. The results showed that xylem tissue size was closely correlated with ground tissue size isometrically across species, while undergoing significant y- or/and x-intercept shift in response to altitudinal belts. Further, a conspicuous characteristic of terminal shoot was its allocation of contrasting tissues between primary xylem and ground tissues with increasing elevation. The result of the PCA showed correlations between anatomical variation, species growth form/height classes and environment.

4. The current study presents a comparative assessment of the allocation of tissue in terminal shoot across phylogenically and ecologically diverse species, and analyzes tissue, function and climate associations with plant growth forms and height classes among species. The interspecific connection between primary xylem ratio and plant size along an elevation gradient suggests the importance of primary xylem in explaining the treeline formation.

## Introduction

The tree growth form has long been viewed as an integrated ecological strategy for species [1] responding to geographic and ecological mechanisms and biotic and abiotic limits [2]–[6]. For alpine plants specifically, an abundant literature shows decreasing height with increasing altitude because of climate (e.g., [7]–[8]). Xylem properties and evolution may be strongly influenced by changes in climate; therefore, they are believed to be a functional adaptation. For example, anatomical characteristics of wood are thought to be proxies for climate, especially for mean annual temperature [9]. This relationship is likely because cool temperatures associated with higher altitudes inhibit the formation of new tissues or tissue renewal [10], apical meristematic activities [11]–[13], or cambial activities (i.e., xylogenesis, see [14]–[15]).

Plant height is closely linked with plant’s anatomical structure and function, which are, in turn, related to the composition and behavior of plant cells [16]. Woody dicotyledons’ height growth is almost entirely dependent on the height increment of the annual shoot of the current year (defined as ‘terminal shoot’ in the following text) where apical meristematic cells repetitively divide, grow and differentiate. Over time, terminal-shoot tissues accumulate to build a primary structure, which can be divided into three tissue systems: dermal tissue (serving for protection); ground tissue (mainly used for storage); and vascular tissue (included of primary xylem and phloem, regulating support and transport according to the “tissue system” theory [17]). Of the three tissue systems, the primary xylem is a key component of the stem, and its developmental process is prone to be influenced by the environmental conditions that the latter finally imprint on its primary structure [18]–[19]. Increasing tree height is associated with shoot architecture, stem anatomy, and xylem production or allocation [20]–[23]. Based on these observations, the terminal shoots, as basic active modules [24], not only determine the terminal shoots’ vertical growth at the very top of a woody plant but also eventually determine the height of the entire plant, considering the modular structure of vertical growth [17] and the integration among functional modules in the body of woody species [25].

It is often suggested that there are complex tradeoffs among functions delivered by different tissue types for a given amount of tissue (storage vs. support/transport by Poorter et al., 2009 [26]; Ewers’ tradeoff triangle in Baas et al., 2004 [27]; Bazzaz & Grace 1997 [28]). Meanwhile, it has been proposed that there is allometry of modular organisms between the terminal shoot and the whole organism [25], [29]–[30]. With above information, we can infer that the different tissue structures in a terminal shoot would likely be related to the tissue amount in a similar way as reported about cambial activity in shoot [31] when discussing xylary effects on plant height. If so, it can be expected that there would be altitudinal-specific scaling relationships between the primary xylem and the other tissues (e.g. ground tissue). Additionally, it is justifiable to predict that terminal shoots at higher altitudes would add ground tissue rather than xylem tissue to cope with the harsh environments at higher altitudes, accounting for the tendency of storage amounts in the plant body to be higher under cold and arid conditions [32].

Gartner, 1995 [33] argues that plant height is greatly affected by the height-dependent pattern of allocation of support tissue (such as xylem tissue in stem), which is one tradeoff (i.e. between growth and photosynthetic requirements) of the five principal tradeoffs of stem. Taller plants have to allocate more resources to unproductive support tissue (e.g., woody tissues) than the shorter ones [33]–[34]. This prediction has been successfully applied to a few of trees’ strategies for competing for light [35]; it may also hold true for cold conditions (e.g., Körner, 2012 [36]). Attempts at a functional explanation of treeline have explored the potential causes (see the summary of Stevens & Fox, 1991 [37], Körner, 2003 [38], 2012 [36] and their references), uncovering a multitude of factors for treeline formation. One of them is growth limitation hypothesis (Körner, 1998 [10], 2003 [38]), that is, plant growth form near the treeline is limited by the production and differentiation of new cells in growing tree tissue from meristems. This theory emphasizes the connection between plant anatomical structure and stature, i.e. trees wood properties are decisive for tree performance in mountain regions, and has been verified by Rossi et al., 2007 [13] on the cambial activity in conifers relative to altitudinal difference. However, the supporting evidence for the hypothesis at a species level (especially for dicot woody species) is very rare. We believe that the generality of the above theories need further validation when applying them to treeline formation and so are their implications for the studies of treeline ecology, since there are diverse dicot species in alpine regions besides a few conifers.

This study focuses on tissue size (including epidermis, cortex, fiber sheaths, phloem, xylem, pith, and starch sheaths if present) and their allocation within a terminal shoot across 100 woody species living in four climatic zones ranging from 1800–4500 m a.s.l. of Gongga Mountain (both above and below treeline species) in southwest China. We measured tissue area and their proportion of cross-sectional area to a terminal shoot for the focal species because cross-sectional tissue area is considered as a good measure [4]. We analyzed the allometric scaling relationships between xylem tissue and ground tissue of species from four climatic zones to examine the trend of adaptation to altitudinal climate at an interspecific level; then, we assess the relationships between primary xylem and plant growth form/height classes along the altitudinal gradient. Because our study included a wide range of species and growth forms, there is a low likelihood of confounding phylogenetic effects on our findings [39].

## Materials and Methods

### Area description

The sampling sites were located along the eastern slope of Gongga Mountain in the Hailuogou national nature reserve in Sichuan province, southwestern China (29°32′–29°37′N, 101°58′–102°04′E). The mountain rises from 1400 m (a.s.l., at the base) to 7556 m (a.s.l., at the top), with a relative elevation change over 6000 m and thus with changes in climate and in vegetation composition (four distinct vegetation zones) across the altitudinal gradient studied [40].

### Ethics Statement

The field study for each location was allowed to be conducted by the staff of the Gonganshan National Nature Reserve. There was no vertebrate involved in this study.

### Material studied

A total of 100 species were chosen which represented the common or dominant species of the four vegetation types on the eastern slope from 1800 to 4500 m (asl.). There were 3 same species sampled from different altitudes, i.e., *Salix hypoleuca* (3000 m, 4500 m), *Salix luctuosa* (2200 m, 3000 m) and *Spiraea veitchii* (3000 m, 3600 m), respectively. These samples from a range of ecologically and phyletically diverse species including tree height of different orders of magnitude. The species were divided into four groups, A (45 species), B (22 species), C (20 species) and D (16 species) by altitudinal belt from low to high, respectively (Table 1 and Table 2; varieties and subspecies were not included in the Table1). Group D was sampled from above the treeline, contained only of shrubs (Table 1). The 100 species belong to 47 genera in 23 families of which 14 have compound leaves.

**Table 1:** Trait means within terminal shoot for the 100 woody broad-leaved species on Gongga Mountain, southwestern China, in which 103 species samples are included.

Species	Family	Altitude(m a.s.l.)	Growth form/height classes	Terminal shootarea (mm^2^)	Ground tissuearea (mm^2^)	Vascular tissuearea (mm^2^)	Xylemarea (mm^2^)	Pitharea (mm^2^)
*Tetracentron sinense*	Tetracentraceae	1800	LT	5.741	3.552	1.279	0.881	0.634
*Populus lasiocarpa*	Salicaceae	1800	MT	6.907	4.191	2.433	1.384	1.255
*Populus purdomii*	Salicaceae	1800	LT	7.752	5.223	2.243	0.740	1.747
*Betula albo-sinensis*	Betulaceae	1800	LT	0.840	0.322	0.436	0.217	0.129
*Vaccinium sprenglii*	Ericaceae	1800	LS	5.456	3.588	1.346	0.557	0.403
*Machilus pingii*	Lauraceae	1800	LT	10.301	8.315	1.718	0.732	1.897
*Acer laxiflorum*	Aceraceae	1800	MT	11.861	7.301	4.036	2.174	3.095
*Rhododendron calostrotum*	Ericaceae	1800	MS	3.116	1.750	1.196	0.869	0.460
*Quercus engleriana*	Fagaceae	1800	MT	4.653	2.522	1.741	0.768	0.666
*Rhododendron glaucophyllum*	Ericaceae	1800	MS	6.667	5.308	1.273	0.598	1.901
*Ilex chinensis*	Aquifoliaceae	1800	MT	3.253	1.282	1.855	1.525	0.166
*Carpinus omeiensis*	Betulaceae	1800	ST	1.799	0.589	1.151	0.896	0.209
*Magnolia dawsoniana*	Magnoliaceae	1800	MT	5.333	3.736	1.435	0.711	0.963
*Rhododendron coeloneurom*	Ericaceae	1800	ST	9.005	7.227	1.637	1.088	2.461
*Betula utilis*	Betulaceae	1800	LT	1.073	0.443	0.581	0.317	0.196
*Zanthoxylum schinifolium*	Rutaceae	1800	MS	4.747	3.025	1.650	1.046	1.063
*Cyclobalanopsis gracilis*	Fagaceae	1800	MT	1.115	0.501	0.538	0.232	0.163
*Neolitsea aurata*	Lauraceae	1800	MT	3.013	1.776	1.111	0.592	1.239
*Phoebe faberi*	Lauraceae	1800	MT	3.592	1.437	1.945	1.406	0.631
*Acer sinense var. concolor*	Aceraceae	1800	LS	2.251	1.340	0.851	0.587	0.904
*Berchemia sinica*	Rhamnaceae	1800	LS	2.106	1.028	1.078	0.502	0.359
*Rosa omeiensis*	Rosaceae	1800	LS	1.775	0.956	0.819	0.407	0.307
*Acer ginnala*	Aceraceae	1800	ST	2.161	1.137	1.024	0.513	0.396
*Sorbus rehderiana*	Rosaceae	1800	ST	1.094	0.660	0.434	0.174	0.186
*Acer maximowiczii*	Aceraceae	1800	MT	2.815	1.558	1.257	0.665	0.803
*Tetradium glabrifolium*	Rutaceae	1800	MT	9.290	6.537	2.754	1.468	2.002
*Pyrus pashia*	Rosaceae	1800	MT	2.107	0.892	1.216	0.727	0.245
*Salix phanera*	Salicaceae	1800	LS	9.218	4.851	4.368	2.273	1.619
*Maddenia hypoxantha*	Rosaceae	1800	ST	2.061	1.178	0.883	0.443	0.640
*Meliosma myriantha*	Meliosmaceae	1800	MT	1.684	1.031	0.653	0.320	0.367
*Pyracantha fortuneana*	Rosaceae	1800	LS	3.014	1.500	1.515	0.838	0.597
*Ilex macrocarpa*	Aquifoliaceae	1800	MT	1.056	0.743	0.313	0.121	0.267
*Pterocarya stenoptera*	Juglandaceae	1800	LT	2.840	1.518	1.322	0.626	0.379
*Eleutherococcus gracilistylus*	Araliaceae	1800	ST	3.522	2.539	0.983	0.446	1.075
*Deutzia longifolia*	Hydrangeaceae	1800	LS	1.570	0.920	0.650	0.320	0.296
*Corylopsis willmottiae*	Hamamelidaceae	1800	LS	2.718	1.296	1.422	0.709	0.516
*Quercus cocciferoides*	Fagaceae	1800	MT	2.576	1.932	0.644	0.332	0.572
*Sorbus hemsleyi*	Rosaceae	1800	MT	1.776	0.883	0.893	0.608	0.277
*Ulmus bergmanniana*	Ulmaceae	1800	LT	0.838	0.537	0.301	0.164	0.163
*Viburnum hanceanum*	Viburnaceae	1800	MS	0.801	0.522	0.279	0.122	0.097
*Meliosma pinnata*	Meliosmaceae	1800	ST	7.208	3.476	3.732	2.510	1.765
*Sorbus pohuashanensis*	Rosaceae	1800	ST	1.665	1.015	0.650	0.308	0.515
*Elaeagnus angustata*	Elaeagnaceae	1800	LS	12.051	7.927	4.124	2.016	3.094
*Viburnum cinnamomifolium*	Viburnaceae	2200	ST	7.224	5.027	2.197	1.296	1.756
*Litsea cubeba*	Lauraceae	2200	MT	2.296	1.697	0.543	0.310	1.054
*Acer flabellatum*	Aceraceae	2200	MT	7.970	3.387	4.435	3.168	1.465
*Eurya chinensis*	Theaceae	2200	LS	3.058	1.496	1.035	0.615	0.470
*Cerasus dielsiana*	Rosaceae	2200	MT	4.124	1.705	1.760	1.124	0.731
*Lindera aggregata*	Lauraceae	2200	LS	2.333	1.459	0.830	0.339	0.851
*Rhododendron argyrophyllum*	Ericaceae	2200	ST	5.692	4.465	1.101	0.525	1.437
*Cerasus glabra*	Rosaceae	2200	LS	0.875	0.505	0.320	0.154	0.078
*Sorbus pallescens*	Rosaceae	2200	ST	3.510	2.497	0.774	0.484	0.548
*Rhamnus hemsleyana*	Rhamnaceae	2200	ST	5.379	3.277	1.975	1.170	1.122
*Hydrangea strigosa*	Hydrangeaceae	2200	LS	13.486	9.760	3.397	2.151	8.057
*Acer davidii*	Aceraceae	2200	MT	3.926	2.646	1.209	0.463	1.211
*Rhododendron oreodoxa*	Ericaceae	2200	ST	2.608	1.828	0.709	0.412	0.871
*Carrierea calycina*	Flacourtiaceae	2200	MT	4.760	2.417	2.065	1.054	0.489
*Salix luctuosa*	Salicaceae	2200	ST	1.499	0.934	0.515	0.225	0.291
*Skimmia japonica*	Rutaceae	2200	MS	4.612	3.544	0.853	0.568	0.686
*Ilex yunnanensis*	Aquifoliaceae	2200	MT	0.869	0.674	0.147	0.062	0.219
*Salix cathayana*	Salicaceae	2200	MS	3.304	1.913	0.970	0.397	0.420
*Meliosma cuneifolia*	Meliosmaceae	2200	MT	2.782	1.604	1.177	0.588	0.420
*Tetradium ruticarpa*	Rutaceae	2200	LS	2.162	0.755	1.407	0.906	0.261
*Enkianthus deflexus*	Vacciniaceae	2200	ST	4.388	2.619	1.769	1.011	1.332
*Maddenia hypoxantha*	Rosaceae	2200	ST	4.332	2.775	1.557	0.564	0.765
*Ribes moupinense*	Grossulariaceae	2200	LS	3.113	2.146	0.967	0.366	0.498
*Pterocarya hupehensis*	Juglandaceae	2200	MT	1.416	0.860	0.557	0.231	0.258
*Acer caudatum*	Aceraceae	3000	MT	6.611	4.645	1.588	0.482	3.337
*Rhododendron kangdingense*	Ericaceae	3000	LS	3.516	2.371	1.064	0.706	0.781
*Rhododendron maculiferum*	Ericaceae	3000	MS	9.553	6.573	2.846	2.038	2.075
*Euonymus alatus*	Celastraceae	3000	LS	1.577	1.009	0.475	0.251	0.130
*Sorbus prattii*	Rosaceae	3000	LS	27.641	22.550	4.642	2.652	5.488
*Cerasus clarofolia*	Rosaceae	3000	MT	1.290	0.631	0.643	0.420	0.274
*Viburnum dilatatum*	Viburnaceae	3000	LS	3.951	2.884	0.916	0.612	1.147
*Malus prattii*	Rosaceae	3000	MT	4.786	2.928	1.607	0.451	0.878
*Lonicera tangutica*	Caprifoliaceae	3000	MS	1.826	1.100	0.561	0.174	0.116
*Ribes tenue*	Grossulariaceae	3000	LS	1.322	0.950	0.322	0.151	0.256
*Deutzia setchuenensis*	Hydrangeaceae	3000	MS	1.242	0.674	0.457	0.291	0.332
*Euonymus porphyreus*	Celastraceae	3000	LS	1.399	1.103	0.225	0.135	0.133
*Salix luctuosa*	Salicaceae	3000	LS	0.823	0.481	0.288	0.092	0.122
*Spiraea veitchii*	Rosaceae	3000	LS	0.855	0.478	0.288	0.148	0.224
*Salix hypoleuca*	Salicaceae	3000	LS	1.470	0.888	0.528	0.131	0.210
*Cotoneaster bullatus*	Rosaceae	3000	MS	3.059	1.872	1.077	0.595	0.696
*Ribes glaciale*	Grossulariaceae	3000	LS	3.782	2.397	1.290	1.066	1.638
*Gaultheria veitchiana*	Vacciniaceae	3000	SS	1.872	1.335	0.487	0.257	0.351
*Gaultheria nummularioides*	Vacciniaceae	3000	SS	0.757	0.522	0.188	0.111	0.083
*Sorbus multijuga*	Rosaceae	3000	ST	3.240	1.730	1.510	0.843	0.903
*Spiraea veitchii*	Rosaceae	3600	LS	1.051	0.748	0.285	0.117	0.367
*Spiraea alpina*	Rosaceae	3600	MS	1.087	0.616	0.329	0.209	0.351
*Sorbus hupehensis*	Rosaceae	3600	MT	7.855	5.178	1.469	0.648	1.025
*Vaccinium sikkimense*	Ericaceae	3600	MS	3.361	2.467	0.734	0.472	0.678
*Rhododendron dendrochairs*	Ericaceae	3600	MS	1.925	1.242	0.653	0.468	0.330
*Rhododendron orbiculare*	Ericaceae	3600	LS	17.480	14.856	2.481	1.621	2.767
*Ribes vilmornii*	Grossulariaceae	3600	LS	3.391	2.035	1.194	0.626	0.686
*Rhododendron concinnum*	Ericaceae	3600	LS	3.121	2.248	0.811	0.526	0.881
*Ribes tenue*	Grossulariaceae	4500	SS	2.744	2.093	0.593	0.426	0.654
*Salix spathulifolia*	Salicaceae	4500	SS	3.604	2.839	0.491	0.172	0.804
*Salix hypoleuca*	Salicaceae	4500	MS	3.931	2.195	1.632	1.021	0.889
*Pentaphylloides glabra*	Rosaceae	4500	MS	0.642	0.341	0.242	0.072	0.218
*Lonicera japonica*	Caprifoliaceae	4500	SS	4.069	2.094	1.888	0.944	0.944
*Salix flabellaris*	Salicaceae	4500	SS	1.126	0.892	0.201	0.039	0.165
*Salix souliei*	Salicaceae	4500	SS	0.559	0.459	0.081	0.021	0.029
*Cassiope selaginoides*	Vacciniaceae	4500	SS	0.236	0.133	0.073	0.024	0.079

Ground tissue area is the sum of cortex and pith area; vascular tissue area is the sum of xylem and phloem area. The classification of growth form and height class is defined by reference to Song, 2001[48] and Moles et al., 2009 [49]. Ss  =  small shrub (<0.5 m), Ms  =  middle shrub (0.5∼2 m), Ls  =  large shrub (2∼5 m), St  =  small tree (5∼8 m), Mt  =  middle tree (8∼25 m), Lt  =  large tree (>25 m).

**Table 2:** Summary of standardized major axis regression analyses for ground tissue area and xylem tissue area at four altitudinal sites on Gongga Mountain, southwestern China.

Group	Altitude (m a.s.l.)	n	r^2^	p	y-intercept	Slope	Low CI	Upp CI
A	1800≤a.s.l.<2200	45	0.527	<0.001	–0.461	0.854	0.692	1.054
B	2200≤a.s.l.<3000	22	0.490	<0.001	–0.572	1.268	0.914	1.759
C	3000≤a.s.l.<3600	20	0.736	<0.001	–0.689	0.987	0.767	1.269
D	3600≤a.s.l.<4500	16	0.694	<0.001	–0.761	1.213	0.888	1.656

A, B, C and D  =  corresponding species group respectively; n  =  the number of species included; CI  =  the confidence interval, Low CI does 95%  =  confidence lower limit, Upp CI  = 95% confidence upper limit.

Species Group A, sampled from the evergreen/broad-leafed forest zone (1800–2200 m), was mainly composed of the Lauraceae, Fagaceae, and Theaceae species. The zone has the warmest temperatures (annual effective accumulated temperature ≥10°C is *c*. 2500–3800°C) of the four sites studied and has moderate rainfall (mean annual rainfall 1000–1600 mm). Species Group B was from the coniferous and deciduous mixed forest zone (2200–3000 m) dominated by *Betula* spp., *Acer* spp., *Tetracentron sinense*, *Cercidiphyllum japonicum*, *Euptelea pleiospermum*, *Picea brachytyla*, *Sorbus astateria* and *Rhododendron calostrotum* [41]. It is in an area of sustained cloud cover, with a mild climate and the greatest rainfall of the four sites (mean annual rainfall 1600–1800 mm). Species Group C was gathered in the subalpine coniferous forest zone (3000–3600 m) growing common species including *Tsuga* spp., *Picea* spp., *Abies fabric*, and *A. faxoniana*. This zone is cold and damp, and its upper margin is a transitional ecotone from tree to shrub with a lower margin of treeline approximately 3600 m [42]. The annual effective accumulated temperature (≥10°C) is *c*. 200–1500°C and the average annual rainfall is 1500–1600 mm. Species Group D was from the alpine shrub zone (3600–4500 m), dominated by *Rhododendron* spp., *Spiraea* spp., *Ribes* spp., and *Salix* spp. [42]–[43]. This zone is the only one vegetation belt above the treeline of the study sites. It is severe cold, windy, mean annual temperature <0°C; the snow-covered duration is as long as half year, and generally unfavorable for plant growth.

Terminal shoot samples were collected from July to August when they were fully expanded and mature, and intact and healthy.

### Methods and Techniques

After a terminal shoot was clipped off, it was fixed and stored in plastic bottles filled with FAA solution (90% 0.5 alcohol+5% glacial acetic acid+5% 0.38 formaldehyde) before the shoot was sectioned. The middle segment of a shoot was cut to make frozen cross-sections. One to five sections per shoot (typically three) per species were photographed [28] under a binocular microscope (Motic BA-300) linked to a digital camera (Moticam 2306, 3.0 M pixel). The scale of the photos was calibrated using a slide-mounted micrometer. Section photos were digitized and analyzed with ImageJ software (version 1.44; ImageJ website. Available: http://imagej.nih.gov/ij/. Accessed 2013 Mar 22).

**Figure 1:**
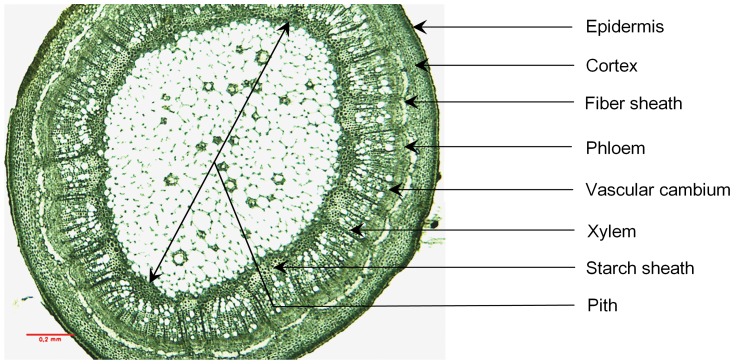
A terminal shoot cross-section. A photograph of *Acer sinense* var. *concolor* showing a typical anatomical structure of a terminal shoot.

The parameters measured were tissue cross-sectional area (i.e., size) of a terminal shoot, including the terminal shoot cross-sectional area (TA), dermal area (DA), cortex area (CA), fiber sheath area (FSA, around the vascular cylinder, if present), phloem area (PHA), xylem area (XA), pith area (PA) and starch-sheath around pith (SS, if present; see Figure 1). The ground tissue area (GA) was the sum of CA and PA. The ground tissue area ratio (GR) was the ratio of GA to TA, and the xylem ratio (XR) was the ratio of XA to TA. Likewise, the vascular tissue area (VA) was the sum of XA and PHA, and the vascular tissue ratio (VR) was the ratio of VA to TA. Variation in tissue size among species is substantial (Table 1). Growth form and height class data were collected for most species from the scientific database of Chinese plant species (DCP; Available: http://db.kib.ac.cn/eflora/Default.aspx. Accessed 2013 Mar 22) and a small number (absent from the DCP database) were gathered in the field during this study. Species’ growth form was a continuous range of height classes (Table 1). Terminal shoot cross-sectional area and the fraction of primary structure to terminal shoot area were calculated from the data collected for this study.

### Data analyses

Trait values were averaged across the 1–5 (mostly three) sections measured per terminal shoot for each of the study species. These values were log_10_-transformed to achieve normal data distributions for analysis, except for ratio values. Type II regression protocols were used to test trait pair scaling relationships. The slopes were calculated as reduced major axes (RMA; [44]), and their confidence intervals were calculated following Pitman, 1939 [45]. Differences in regression slopes among species groups were tested for significant differences according to Warton & Weber, 2002 [46]. Variations in the shift along the common slope of the y-intercept (xylem tissue size) and x-intercept (ground tissue size) were examined by (S)MATR [47] and ANOVA with post hoc Turkey tests.

We also evaluated the covariance among plant growth form/height class of species, shoot tissue anatomical traits, and altitudes of 103 woody species samples with a PCA (using Canoco 4.5), and bivariate correlation regression across species, where the species were grouped into six height classes (c.f. Song, 2001 [48] and Moles et al., 2009 [49]). Prior to PCA, a DCA (detrended correspondence analysis) was completed to determine whether a unimodal or a linear method should be used following Leps & Smilauer, 2003 [50]. The lengths of the gradient (in terms of Canoco software) were found to be smaller than 3 after the DCA, so PCA was applicable. One-way ANOVA for the independent variables was conducted to detect trait differences among species groups. These analyses were performed using STATISTICA [51].

## Results

### The scaling relationships between xylem tissue vs. ground tissue size among species groups

**Figure 2:**
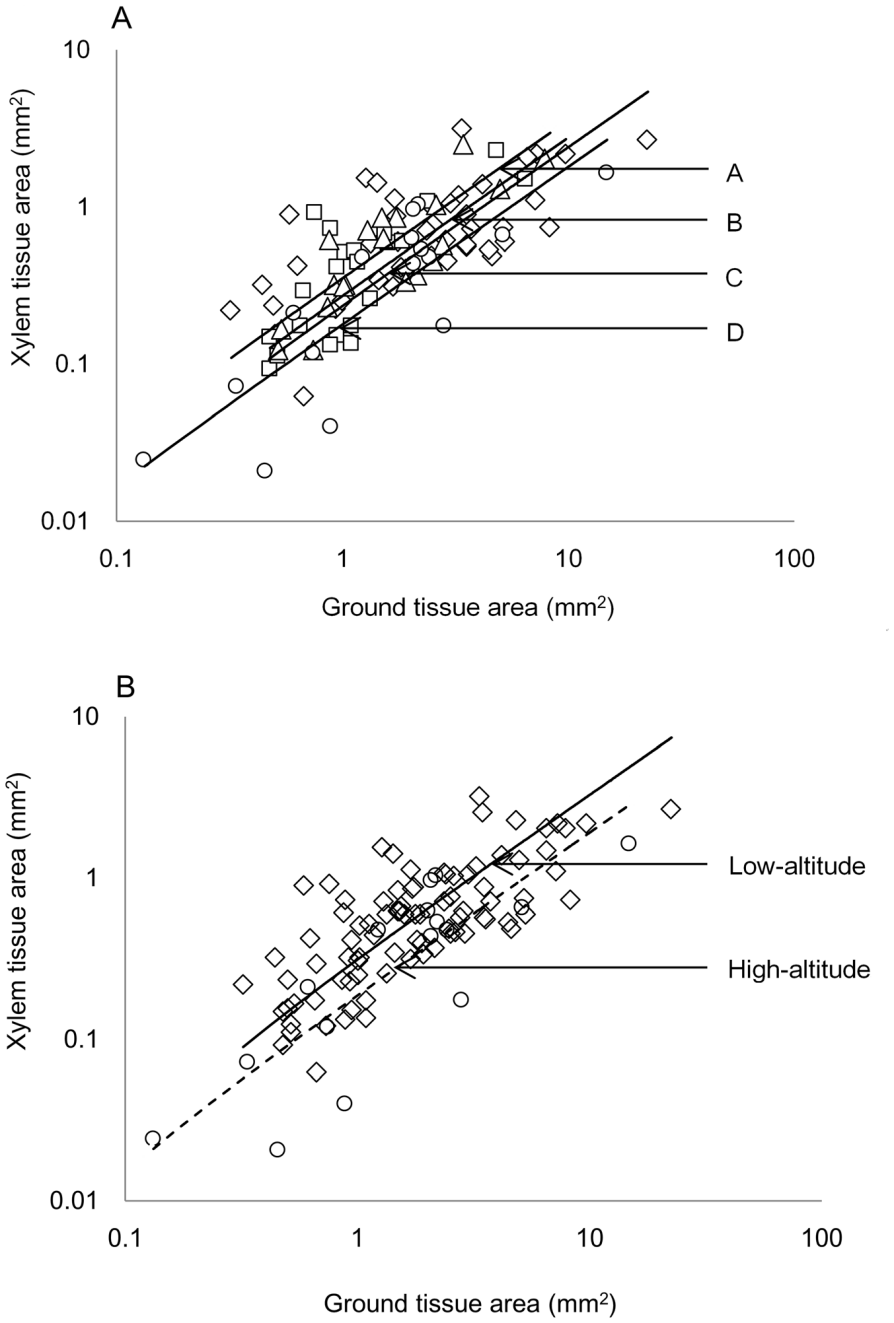
The relationship between xylem tissue area and ground tissue area. Cross-species bivariate relationship plot for xylem tissue vs. ground tissue area for the woody species grouping along an altitudinal gradient on Gongga Mountain, southwestern China. There are regression lines (slope  =  common slope) of the reduced major axis (RMA) in the graph for the (A) four species groups which are labeled in order A, B, C and D respectively. Legends: diamonds  =  Group A; squares  =  species Group B; triangles  =  species Group C and circles  =  species Group D; (B) two species groups at low (66 species from below 3000 m, diamonds, full line) and high altitude (36 species from above 3000 m, circles, broken line).

The four species groups shared a common slope for the scaling relationships between xylem area and ground tissue area (slope  =  1.014, 95% CI =  [0.880, 1.155]; r^2^ = 0.567, *p* <0.001); it did not depart from 1 (*p* = 0.929) and was independent of species group (*p* = 0.125; Figure 2A; Table 2). The isometric association was also conserved across species within each site (Table 2). Further, we found a significant shift between elevations in the four groups (*p* = 0.003), and the y-intercept values were decreasing with elevation (A>B>C>D, Figure 2A; Table 2). In addition, the elevation shift in the common slope of the scaling relationship was significantly different (*p* = 0.001) between the high altitude group (36 species, Groups D and C; r^2^ = 0.698, *p*<0.001, slope = 1.123, 95% CI  =  [0.928, 1.359]; y-intercept  = –0.711) and the low altitude groups (66 species, Groups A and B; r^2^ = 0.485, *p*<0.001, slope  = 0.967, 95% CI  =  [0.810, 1.154]; y-intercept  = –0.485). These results show that for a given amount of ground tissue, the higher altitude species generally have less xylem tissue.

Moreover, there was also a significant shift along the common slope (x-intercept; *p* = 0.036) between high and low elevation groups, stressing that ground tissue size was larger in high altitude species than in low altitude ones (Figure 2B).

### Variations in xylem ratio and ground tissue ratio with the increase in altitude

**Figure 3:**
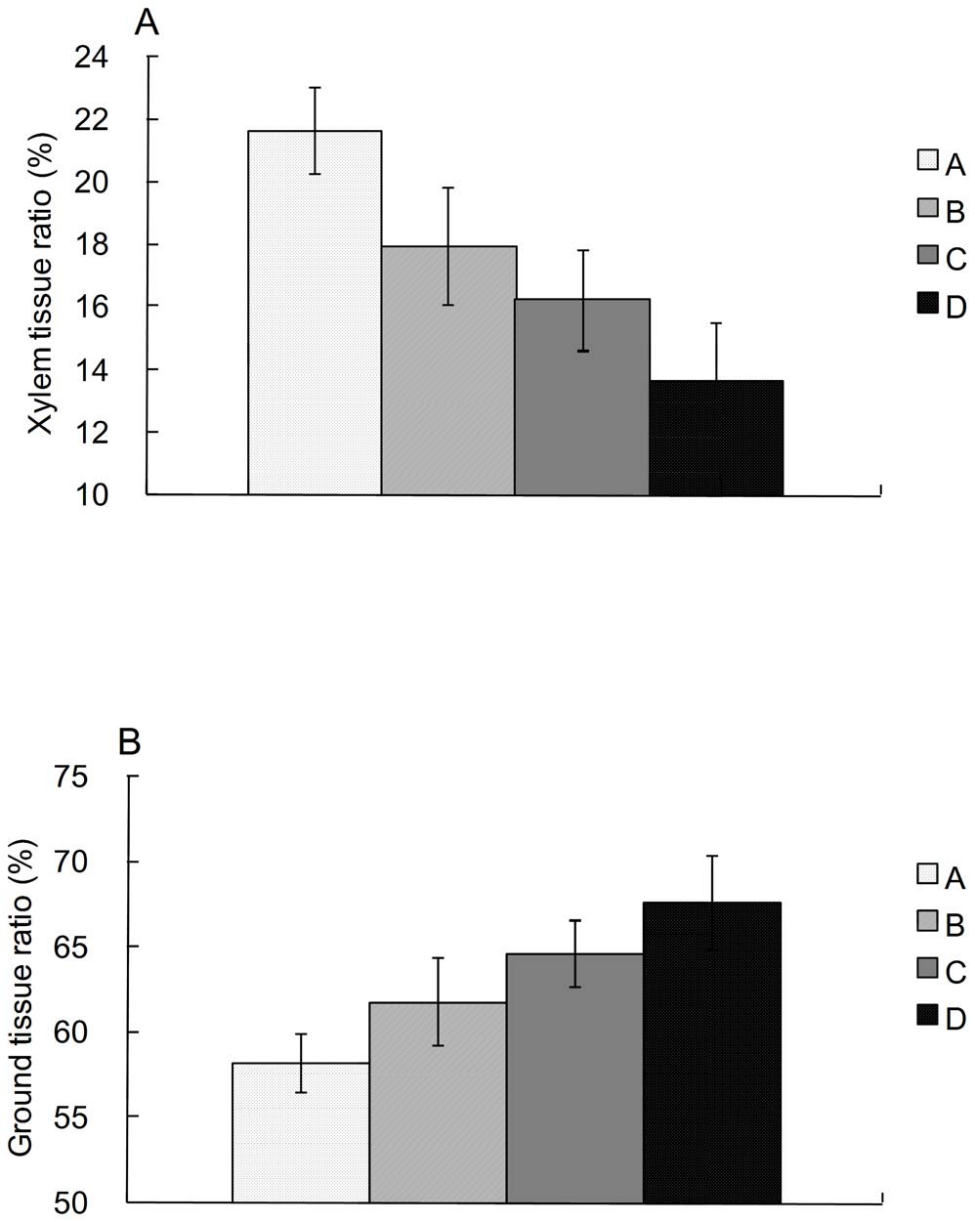
The tissue ratio for species groups. The tissue area to terminal shoot cross-sectional area ratio for species groups varying with an altitude gradient of Gongga Mountain. (A) is Xylem tissues ratio and (B) is ground tissue ratio. A, B, C and D =  corresponding species groups respectively.

Not surprisingly, the xylem ratio (XR) declined with increasing altitude in stages (ANOVA, F = 4.278, *p* = 0.007; Figure 3A), with the average percentage nearly declining by half from Group A (21.7%) to Group D (13.7%), while the XR for Group A was significantly higher than for Groups C and D (*p*<0.05, ANOVA post hoc test). Likewise, the GR rose significantly (ANOVA, F = 3.476, *p* = 0.012; Figure 3B), with an increase of 9.4%. These results together show that the XR has a gradual downward trend with increasing altitude while GR has the opposite trend. The higher-altitudinal species have less woody tissue in terminal shoots but more ground tissues than the lower species. In addition, the XR of the 2 of the 3 same species decreased from low to high sites (i.e., from 15.02% at 2200 m to 11.16% at 3000 m in *Salix luctuosa*, from 17.35% at 3000 m to 11.17% at 3600 m in *Spiraea veitchii*, respectively), generally consistent with the varying trend of XR; but the XR of *Salix hypoleuca* increased from 8.89% at 3000 m to 25.97% at 4500 m.

### The associations of growth form/height classes, anatomical traits and altitude

**Figure 4:**
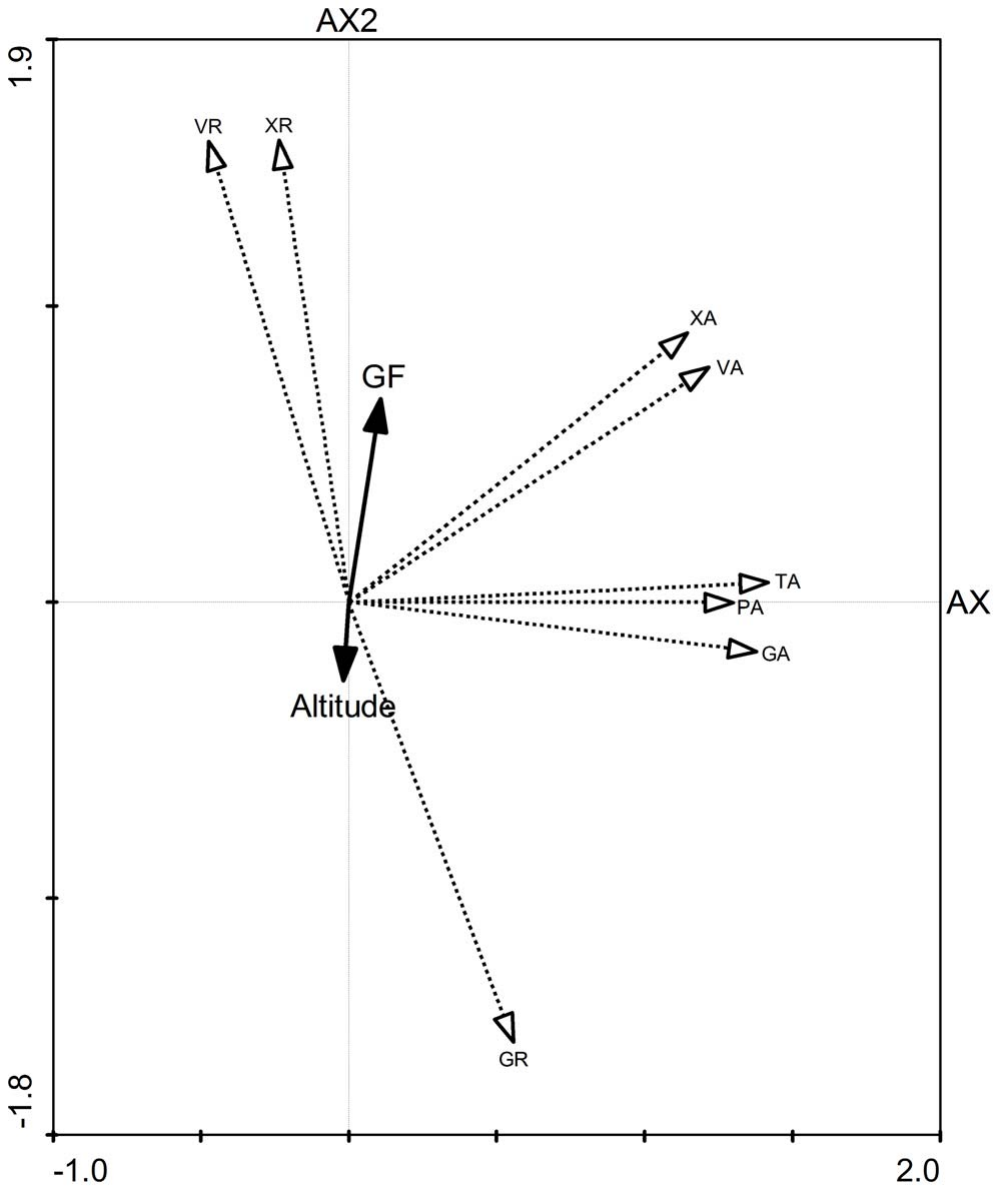
The biplot of PCA. Association among tissue distribution in terminal shoot, growth form/height classes and altitude for 100 woody forest species on Mt. Gongga in southwest China. In the biplot, the horizontal and vertical axes (Ax1 and Ax2) denote the first and the second ordination axis of PCA, respectively. Tissue traits, altitude and species growth form are shown in the diagram. GF  =  growth form/height class, XA  =  xylem tissue area, VA  =  vascular tissue area, TA  =  terminal shoot cross-sectional area, PA  =  pith area, GA  =  ground tissue area, GR  =  ground tissue ratio, VR  =  vascular tissue ratio, XR  =  xylem tissue ratio.

PCA revealed that the first two components accounted for 82.3% of the total variation among the nine traits studied (Figure 4). The first axis (Ax1) explained 47.7% of variance, in the direction of variables TA, GA, VA, PA and XA (Figure 4). The second axis (Ax2) explains 34.6% of the variation, in the direction of three ratio variables VR, XR, GR, as well as species growth form/height classes (GF, in Figure 4) and the environmental variable (Altitude, in Figure 4), where species growth form and altitude were much closer to the second axis than the first axis. In addition, the first axis explains only 0.4% of the variation in species-environment relationship, but the second axis explains 78.6%.

These results indicate that the second axis most likely represents the species-environment relationship or the relationship of growth form/plant height, anatomical allocation, and altitude correlation (Figure 4). The XR has the smallest angle to GF while TA, PA and GA has much bigger ones (Figure 4), indicating that the XR may have the closest connection to plant size, but TA, PA and GA the least. In addition, Figure 4 shows that species height is positively related to XR, but negatively related to altitude and GR.

## Discussion

As expected, there are significant interspecific variations in the relative proportion of conducting and storage tissues in response to environmental stress, while the exponent value for tissue scaling does not vary significantly. There are also ecological correlates between these variations and species growth forms/height class. These results partly confirm the predictions of Gartner, 1995 [33] and Körner, 1998 [10], 2003 [38] as applied to woody plant stems. Moreover, since the present study was conducted among species across four vegetation zones, the interspecific tissues-partitioning patterns observed in dicot woody species may be of more general significance to treeline formation.

### 1) The consistent scaling of xylem tissue size vs. ground tissue size

Primary xylem size increases proportionally with ground tissue size, independent of growth form and even of altitude, demonstrating that there is a scaling relationship between primary xylem size and ground tissue size. Because the slopes are not significantly different from 1, the ratio of xylem tissue and ground tissue does not change with terminal shoot area. Possible explanations of this finding have been discussed in the literature: the division of primary cells and the elongation and differentiation of procambium and ground meristem, is potentially subjected to a “developmental constraint” [52]–[53] or “juvenile phase,” as noted by Rowe & Speck, 2005 [54]. These ideas suggest cell differentiation in early developmental stages is only slightly affected by environment so that their daughter cells are more homologous, although there is evidence that there are notable changes in secondary xylogenesis associated with the environment [13], [15], [55].

### 2) The reduced xylem ratio in stem

Nevertheless, although there is a consistent scaling relationship in xylem and ground tissue area, their y-intercept decreases significantly with altitude. Xylem quantity declines significantly, whereas ground tissue increases along an elevation gradient; thus, lower altitude species have larger xylem than higher ones. The reason for this “counter-balance” pattern may be because of the tradeoff among tissue types in the shoot. Lower altitude terminal shoots invest in relatively smaller ground tissues, which causes larger xylem with increasing altitude and vice versa, thus showing the tradeoff between ground and xylem tissue [28] (there was also the tradeoff in this study, *p*<0.001). And the same trend conserved in 2 of the same species across belts; yet the other one (*Salix hypoleuca*) did not. This inconsistency presumably resulted from that the *Salix hypoleuca* is somewhat special since it is the sole species being able to grow across the whole sample area (1800 m ∼ 4500 m) in the 100 species studied. Maybe, it is a natural phenomenon worth further exploring although it is hard to be explained with the current data.

One of the direct consequences of smaller xylem is the weakened supporting/transporting capability of a young stem. The declining trend of xylem means the terminal shoots near the treeline have to support their appendages using less woody tissue. Without sufficient xylem tissue, higher altitude terminal shoots may fail to support the shoot weight and hardly attain full vertical growth, thus affecting the total plant height [56]. Similar ideas obtained previously that there is a decreased ratio of latewood in stems for *Picea* under colder conditions [31] or *Picea* at higher altitudes due to the suppressed xylogenesis [14], and disproportionally decreased xylem for many shorter herbaceous species [57], indirectly supporting our finding.

### 3) The increased ground tissue allocation in stem

Plants invest more ground tissues in shoots as elevation rises was observed in the current study, revealing the high needs for ground tissues for plants in alpine region. It is comparable with a finding described by Körner, 2003 [38] stated that higher-altitudinal species have more developed mesophyll tissues in their leaves than lower altitude species. Considering ground tissue is similar to mesophyll tissue (because cortex, pith, and mesophyll tissue all belong to ground tissues following the theory of tissue system), it would make a sense in connection with this result even though stems and leaves are different organs.

There are some apparent benefits for species with larger ground tissues in a stressful environment, due to some unique functions of this tissue type. First, large amount of ground tissues may be advantageous for alpine plants recovering from frequent tissue loss resulted from shoot breakage in the windy weather and snow cover. Hence, they are naturally favored since they are living cells with a potential dividing capacity functioning tissue repair (or tissue renewal) compared to their dead counterparts (i.e. xylem tissues), as previously noted by Sveinbjörnsson, 2000 [58]. Second, the observed allocating pattern of ground tissues may have an important implication for understanding species surviving and reproducing in adverse high altitude habitats. The storage of carbohydrates in the ground tissues is usually responsible for effectively buffering carbon deficiencies [37], [59], helping species survive [60], [37] and make a successful vegetative reproduction [34] in adverse high altitude habitats. Therefore, the ground tissues appear to be a reliable element for plants to mediate the adaptive and developmental relationship among growth, survive and reproduction; that seems to deserve further investigation.

### 4) The relationship of growth form/height classes, anatomical traits, and altitude and its implications for treeline formation

Plant height displays a tight positive relationship with xylem-tissue ratio, as opposed to the ground tissue ratio which increases as altitude increases (Figure 4). These significant associations of size, allocation, and environment relationship with apparent differences between above and below treeline species, together confirms the point that plant anatomical structure and even plant height may be limited by the potential available energy [10], [33], [61]. Further, the result may also provide an anatomical clue about the biophysical determinants of height resulting from tissue-specific mechanics [4], [62].

This study found a result that species invest less xylem tissue in their terminal shoots with increasing elevation, with a consistency to the conclusion that a reduction in unproductive tissues is less favored by natural selection in trees than in dwarf shrubs at the same altitude [34]. It highlights once again that plants optimize their resource allocation [32] with rising altitude by adjusting the proportions of xylem and ground tissue as noted above. Accordingly, the partitioning pattern between storing and conducting tissue might become one of the determinants that many bigger woody trees cannot live above a specific altitude (e.g. 3600 m a.s.l. in this study), thus forming treeline from a view of landscape.

## Conclusion

Using a relatively large anatomical dataset of diverse woody dicotyledons, we found that there are tissue sizes or ratio driven processes [63] within stems of woody species growing at different altitudes, implying an ecological strategy in tree in response to distinct environments. The interspecific pattern of evolution in tissue size in this study has not been shown before. These results maybe provide a new functional interpretation of treeline formation resulting from a stem tissue based limit to plant size. The differing tissue structure and function at site-specific altitudes could be one of biological mechanisms in creating patterns of treelines, contributing to the knowledge of phenology and treeline ecology.

## References

[1] Petit RJ, Hampe A (2006) Some evolutionary consequences of being a tree. Annual Review of Ecology Evolution and Systematics 37: 187–214.

[2] Falster DS, Westoby M (2005) Alternative height strategies among 45 dicot rain forest species from tropical Queensland, Australia. Journal of Ecology 93: 521–535.

[3] Fan Z-X, Zhang S-B, Hao G-Y, Slik JWF, Cao K-F (2012) Hydraulic conductivity traits predict growth rates and adult stature of 40 Asian tropical tree species better than wood density. Journal of Ecology 100: 732–741.

[4] Niklas KJ (2007) Maximum plant height and the biophysical factors that limit it. Tree Physiology 27: 433–440. 17241985 10.1093/treephys/27.3.433

[5] Iida Y, Poorter L, Sterck FJ, Kassim AR, Kubo T, et al (2012) Wood density explains architectural differentiation across 145 co-occurring tropical tree species. Functional Ecology 26: 274–282. 10.1890/11-2173.1 24669729

[6] Kempes CP, West GB, Crowell K, Girvan M (2011) Predicting maximum tree heights and other traits from allometric scaling and resource limitations. PLoS ONE 6(6): e20551 doi:10.1371/journal.pone.0020551. 21695189 10.1371/journal.pone.0020551 PMC3113805

[7] Swenson NG, Weiser MD (2010) Plant geography upon the basis of functional traits: an example from eastern North American trees. Ecology 91: 2234–2241. 20836445 10.1890/09-1743.1

[8] Swenson NG, Enquist BJ (2007) Ecological and evolutionary determinants of a key plant functional trait: wood density and its community-wide variation across latitude and elevation. American Journal of Botany 94: 451–459. 21636415 10.3732/ajb.94.3.451

[9] Baas P, Wheeler E (2011) Wood anatomy and climate change. In: Hodkinson TR, Jones MB, Waldren S, Parnell JAN, editors. Climate change, ecology and systematics. New York: Cambridge university press. pp. 141–155.

[10] Körner C (1998) A re-assessment of high elevation treeline positions and their explanation. Oecologia 115: 445–459. 28308263 10.1007/s004420050540

[11] James JC, Grace J, Hoad SP (1994) Growth and photosynthesis of *Pinus sylvestris* at its altitudinal limit in Scotland. Journal of Ecology: 297–306.

[12] Oberhuber W (2004) Influence of climate on radial growth of *Pinus cembra* within the alpine timberline ecotone. Tree Physiol 24: 291–301. 14704138 10.1093/treephys/24.3.291

[13] Rossi S, Deslauriers A, Anfodillo T, Carraro V (2007) Evidence of threshold temperatures for xylogenesis in conifers at high altitudes. Oecologia 152: 1–12. 17165095 10.1007/s00442-006-0625-7

[14] Petit G, Anfodillo T, Carraro V, Grani F, Carrer M (2011) Hydraulic constraints limit height growth in trees at high altitude. New Phytologist 189: 241–252. 20840508 10.1111/j.1469-8137.2010.03455.x

[15] Rossi S, Morin H, Deslauriers A (2011) Causes and correlations in cambium phenology: towards an integrated framework of xylogenesis. Journal of Experimental Botany. 10.1093/jxb/err423 PMC3295399 22174441

[16] Grierson C, Barnes S, Chase MW, Clarke M, Grierson D, et al (2011) One hundred important questions facing plant science research. New Phytologist 192: 6–12. 21883238 10.1111/j.1469-8137.2011.03859.x

[17] Evert RF (2006) Esau's plant anatomy—Meristems, cells, and tissues of the plant body: their structure, function, and development. Hoboken: A John Wiley & Sons, Inc., Publication.

[18] Fonti P, Jansen S (2012) Xylem plasticity in response to climate. New Phytologist 195: 734–736. 22861186 10.1111/j.1469-8137.2012.04252.x

[19] Die A, Kitin P, Kouame FNg, Van den Bulcke J, Van Acker J, et al (2012) Fluctuations of cambial activity in relation to precipitation result in annual rings and intra-annual growth zones of xylem and phloem in teak (*Tectona grandis*) in Ivory Coast. Ann Bot 110: 861–873. 22805529 10.1093/aob/mcs145 PMC3423803

[20] Osada N, Takeda H, Furukawa A, Awang M (2002) Changes in shoot allometry with increasing tree height in a tropical canopy species, *Elateriospermum tapos* . Tree Physiology 22: 625–632. 12069918 10.1093/treephys/22.9.625

[21] Westoby M, Falster DS, Moles AT, Vesk PA, Wright IJ (2002) Plant ecological strategies: some leading dimensions of variation between species. Annual Review of Ecology and Systematics 33: 125–159.

[22] Niklas KJ (1995b) Size-dependent allometry of tree height, diameter and trunk-taper. Annals of Botany 75: 217–227.

[23] King DA (2011) Size-related changes in tree proportions and their potential influence on the course of height growth. In: Meinzer FC, Niinemets Ü, editors. Size-and age-related changes in tree structure and function. pp. 175–191.

[24] Sprugel D, Hinckley T, Schaap W (1991) The theory and practice of branch autonomy. Annual Review of Ecology and Systematics 22: 309–334.

[25] Pigliucci M, Preston KA (2004) Phenotypic integration: studying the ecology and evolution of complex phenotypes: Oxford University Press, USA.

[26] Poorter L, McDonald I, Alarcon A, Fichtler E, Licona JC, et al (2009) The importance of wood traits and hydraulic conductance for the performance and life history strategies of 42 rainforest tree species. New Phytologist 185: 481–492. 19925555 10.1111/j.1469-8137.2009.03092.x

[27] Baas P, Ewers FW, Davis SD, Wheeler EA (2004) Evolution of xylem physiology. In: Hemsley AR, Poole I, editors. The evolution of plant physiology. London: Elsevier Academic Press. pp. 273–295.

[28] Bazzaz F, Grace J (1997) Plant resource allocation. Academic press.

[29] Ishii H (2011) How Do Changes in leaf/shoot morphology and crown architecture affect growth and physiological function of tall trees? In: Meinzer FC, Niinemets Ü, editors. Size-and age-related changes in tree structure and function. pp. 215–232.

[30] Osada N (2011) Height-dependent changes in shoot structure and tree allometry in relation to maximum height in four deciduous tree species. Functional Ecology 25: 777–786.

[31] Gricar J, Zupancic M, Cufar K, Koch G, Schmitt U, et al (2006) Effect of local heating and cooling on cambial activity and cell differentiation in the stem of Norway spruce (*Picea abies*). Annals of Botany 97: 943–951. 16613904 10.1093/aob/mcl050 PMC2803384

[32] Bloom AJ, Chapin FS, Mooney HA (1985) Resource limitation in plants—an economic analogy. Annual Review of Ecology and Systematics 16: 363–392.

[33] Gartner BL (1995) Plant stems: physiology and functional morphology. Academic Press.

[34] Holtmeier F-K, editor (2009) Mountain timberlines—ecology, patchiness, and dynamics. Berlin: Springer.

[35] Sterck F, Schieving F (2011) Modelling functional trait acclimation for trees of different height in a forest light gradient: emergent patterns driven by carbon gain maximization. Tree Physiology 31: 1024–1037. 21893522 10.1093/treephys/tpr065

[36] Körner C (2012) Alpine treelines: functional ecology of the global high elevation tree limits. Switzerland: Springer Basel.

[37] Stevens GC, Fox JF (1991) The causes of treeline. Annual Review of Ecology and Systematics 22: 177–191.

[38] Körner C (2003) Alpine plant life: functional plant ecology of high mountain ecosystems. Berlin: Springer.

[39] Losos JB (2011) Seeing the forest for the trees: the limitations of phylogenies in comparative biology. The American Naturalist 177: 709–727. 10.1086/660020 21597249

[40] Li G, Yang D, Sun S (2008) Allometric relationships between lamina area, lamina mass and petiole mass of 93 temperate woody species vary with leaf habit, leaf form and altitude. Functional Ecology 22: 557–564.

[41] Cheng G, Luo J (2002) Successional features and dynamic simulation of sub-alpine forest in the Gongga Mountain, China. Acta Ecologica Sinica 22: 1049–1056 (in Chinese)..

[42] Shen ZH, Liu ZL, Wu J (2004) Altitudinal pattern of flora on the eastern slope of Mt. Gongga. Biodiversity Science 12: 89–98.

[43] Liu ZG (1985) Vegetation of Gongga Mountain. Chengdu: Sichuan Science and Technology Press (in Chinese).

[44] Warton DI, Wright IJ, Falster DS, Westoby M (2006) Bivariate line-fitting methods for allometry. Biological Reviews 81: 259–291. 16573844 10.1017/S1464793106007007

[45] Pitman ETG (1939) A note on normal correlation. Biometrika 31: 9–12.

[46] Warton DI, Weber NC (2002) Common Slope Tests for Bivariate Errors-in-Variables Models. Biometrical Journal 44: 161.

[47] Falster DS, Warton DI, Wright IJ (2006) SMATR: standardised major axis tests and routines ver 2. : 0 edhttp://www.bio.mq.edu.au/ecology/SMATR/.

[48] Song Y, C (2001) Vegetation ecology. Shanghai: East China normal university press (in Chinese).

[49] Moles AT, Warton DI, Warman L, Swenson NG, Laffan SW, et al (2009) Global patterns in plant height. Journal of Ecology 97: 923–932.

[50] Leps J, Smilauer P (2003) Multivariate analysis of ecological data using CANOCO. Cambridge university press.

[51] Statsoft I (2001) STATISTICA 6.0. Tulsa, Oklahoma.

[52] Harvey PJ, Pagel MD (1991) The comparative method in evolutionary biology. Oxford: Oxford University Press.

[53] Niklas KJ (2006) A phyletic perspective on the allometry of plant biomass-partitioning patterns and functionally equivalent organ-categories. New Phytologist 171: 27–40. 16771980 10.1111/j.1469-8137.2006.01760.x

[54] Rowe N, Speck T (2005) Plant growth forms: an ecological and evolutionary perspective. New Phytologist 166: 61–72. 15760351 10.1111/j.1469-8137.2004.01309.x

[55] Schweingruber FH (2007) Wood structure and environment. Berlin: Springer-Verlag, Germany.

[56] Turnbull CGN (2005) Plant architecture and its manipulation: Wiley-Blackwell.

[57] Niklas KJ (1995a) Plant height and the properties of some herbaceous stems. Annals of Botany 75: 133–142.

[58] Sveinbjörnsson B (2000) North american and european treelines: external forces and internal processes controlling position. AMBIO: A Journal of the Human Environment 29: 388–395.

[59] Gleeson SK, Tilman D (1992) Plant allocation and the multiple limitation hypothesis. American Naturalist 139: 1322–1343.

[60] Li M, Xiao W, Shi P, Wang S, Zhong YD, et al (2008) Nitrogen and carbon source-sink relationships in trees at the Himalayan treelines compared with lower elevations. Plant, Cell & Environment 31: 1377–1387. 10.1111/j.1365-3040.2008.01848.x 18643956

[61] Rossi S, Deslauriers A, Griçar J, Seo J-W, Rathgeber CBK, et al (2008) Critical temperatures for xylogenesis in conifers of cold climates. Global Ecology and Biogeography 17: 696–707.

[62] Koch GW, Sillett SC, Jennings GM, Davis SD (2004) The limits to tree height. Nature 428: 851–854. 15103376 10.1038/nature02417

[63] Weiner J (2004) Allocation, plasticity and allometry in plants. Perspectives in Plant Ecology, Evolution and Systematics 6: 207–215.

